# Exploring the use of a dance-based exergame to enhance autistic children’s social communication skills in the home and school environments: a feasibility study

**DOI:** 10.1080/20473869.2023.2212985

**Published:** 2023-05-22

**Authors:** Phoebe O. Morris, Edward Hope, Tom Foulsham, John P. Mills

**Affiliations:** 1 School of Sport, Exercise Science and Rehabilitation, University of Essex, Colchester, Essex, UK; 2 Department of Psychology, University of Essex, Colchester, Essex, UK

**Keywords:** autism spectrum disorder, exergaming, dance, physical activity, communication skills, social development

## Abstract

**Purpose:** Autistic individuals often display social-communicative differences affecting aspects of daily living. The present study assessed the feasibility and potential efficacy of a dance-based exergame for enhancing autistic children’s social-communication skills. **Methods:** A mixed method, within-subject, pre-test/post-test study design was employed. Children in their home (*n* = 4; M_age_ = 8.25 years old, SD = 0.50) and school environments (*n* = 31; M_age_ = 10.87 years old, SD = 1.61) participated in a dance-based exergaming intervention (*Just Dance)* for six weeks. **Results:** A positive change in children’s social-communication skills was observed (*p* < 0.01). Results suggest *Just Dance* was easy to implement and enjoyable. **Conclusion:** A larger randomised control trial is warranted to confirm the effectiveness of the dance-based exergame.

Autism Spectrum Disorder (ASD) is a neurodevelopmental condition affecting approximately 1 in every 100 children across the UK (Chiarotti and Venerosi, 2020). It is an incredibly heterogenous condition, where autistic individuals display an array of strengths and challenges across a spectrum of domains; including social, emotional, cognitive, and motor functioning. However, in order to meet the diagnostic criteria for ASD, individuals must experience difficulties with social interaction and communication and express repetitive or intense interests or behaviours (American Psychiatric Association, 2013).

Autistic individuals often display prevalent and chronic social-communicative and emotional differences that can affect various aspects of daily living. For example, young autistic children display challenges with imitation abilities and joint attention skills, which are often indicative of later social development (Charman *et al.* 2003, Dawson *et al.* 2004, Toth *et al.* 2006). Older children on the autistic spectrum may struggle to initiate social bids, engage in reciprocal communication, demonstrate interpersonal synchrony, and initiate or maintain eye contact; thus, restricting social interactions (Matson *et al.* 1990, Matson *et al.* 2007). Often poor social development and limited communication skills can predispose negative outcomes in later life; for example, having smaller social circles, experiencing peer rejection, and increasing the feeling of loneliness (Palmer *et al.* 2016, Wang *et al.* 2018). These negative outcomes can have a detrimental effect on an individual’s quality of life, affecting areas of mental health and well-being, alongside academic achievements and career aspirations (Howlin and Magiati, 2017). Autistica, the UK’s leading autism research charity, identified that increasing provisions for the development of social-communication interventions was one of the autistic community’s top research priorities (Autistica, 2016). Therefore, researchers must aim to support and enhance communication skills and the social development of autistic children, in order to better their quality of life and limit negative outcomes during childhood and further into adulthood.

## Exergames, mirroring, and rhythm – JUST DANCE

In addition to the core social traits associated with autism, autistic individuals often have sensory and motor differences that may further affect their emotional regulation, social engagement, and development of social expertise (Bhat *et al.* 2011, MacDonald *et al.* 2013, Marsh *et al.* 2009, Robledo *et al.* 2012). Such motor challenges may restrict an autistic individual’s ability to initiate or maintain joint attention and achieve interpersonal synchrony, which are often essential features of effective social communication - coordinating actions in time with a social partner (Bhat *et al.* 2011, Dadgar *et al.* 2017, Fulceri *et al.* 2018). Therefore, supporting and enhancing motor proficiencies may lessen the cognitive demand needed for social interactions for autistic individuals, enhancing their perceived social skills (Huang *et al.* 2020, Mastrominico *et al.* 2018, Najafabadi *et al.* 2018). Embodiment theories offer a plausible explanation for how increased motor activities may improve social functioning, arguing that cognition (including social cognition) and emotion are both grounded in bodily states (Eigsti, 2013). Thus, body-based or movement-based interventions can foster embodied experiences, positively influencing social cognition and development.

One such intervention, grounded in the concept of embodiment, is dance movement therapy (DMT). DMT has previously been used within the autistic population to promote expression (Hildebrandt *et al.* 2016, Koch *et al.* 2015, Koehne *et al.* 2016), psychological well-being (Aithal *et al.* 2021), and communication and social interaction (DeJesus *et al.* 2020), albeit with minimal effect on empathy (Chen *et al.* 2022). It aims to utilise rhythmic bodily movements and mirroring to explore sensory and motor experiences; developing bodily awareness, expressivity in movement, and enhance the ability to synchronise interpersonally. Subsequently, enhancing both social competency and emotional regulation, alongside naturally occurring motor proficiencies; traits that are commonly affected in autism (Koch *et al.* 2015, Takahashi *et al.* 2019). Despite the observed positive effects of DMT, accessing it can be difficult for many autistic individuals as it involves travelling to various locations, the presence of a trained professional, unfamiliar environments, and substantial therapy costs – typifying the downsides of many current social-communication interventions for autistic children. Therefore, ways to implement effective elements of DMT; such as rhythmic movements and mirroring, into more accessible interventions are needed for the autistic population to access its potential benefits for social communication skills (Morris *et al.* 2021).

Exergames are exercise-inducing videogames that span a range of different sports and physical activities and can be played in a variety of locations, including both the home and school environment. Furthermore, social exergaming not only encourages motor movements but also provides a valuable platform for social exchanges between individuals playing each game (Marker and Staiano, 2015, Rüth and Kaspar, 2021). For example, various exergames played in both the home and school environment have been observed to foster social motivation, game continuance, and self-efficacy (Marker and Staiano, 2015), as well as increase prosocial behaviours and group cohesion between typical-developing pupils (Quintas *et al.* 2020, Rüth and Kaspar, 2020). Yet, these benefits have not been widely explored in neurodivergent populations, warranting investigation of the feasibility of exergames for autistic children. One widely recognised exergame, known as *‘Just Dance’,* induces rhythmical motor movements to external stimuli and involves mirroring a dancing avatar on the screen. Participants also engage in interpersonal motor synchrony whilst playing the dance-based exergame. As such, the use of *Just Dance* may be an accessible intervention that incorporates the valuable elements of DMT to enhance autistic children’s social communication skills. Therefore, the present study explores the feasibility of a dance-based exercise videogame, namely *Just Dance*, as a vehicle to enhance autistic children’s social communication skills. The action and practice of engaging in mirroring, attending to rhythmical stimuli, and sharing interpersonal synchrony through the use of *Just Dance* is of significance for this particular study, rather than explicit game-based learning.

## Delivering an intervention in multiple locations

Currently, many of the therapeutic interventions that target communication skills and social development require vigorous training to be delivered by qualified professionals in an unfamiliar and single setting. This subsequently limits the accessibility of many interventions for autistic individuals due to travel restrictions, financial costs, and excessive programme durations (Lee and Meadan, 2021, Raymaker *et al.* 2017, Tek and Landa, 2012, Wainer and Ingersoll, 2015). Likewise, relying on the availability of a trained professional often incurs long waitlist times, where diffident social behaviours can sometimes deteriorate (Myers *et al.* 2018). As a result, there is a clinical and social need to develop low-cost interventions that are accessible to geographically dispersed participants or those in remote locations, that do not rely on face-to-face or in-person participation and do not always require the presence of a trained professional (Lee and Meadan, 2021). Furthermore, research favours interventions that can be delivered across multiple settings; such as teacher- and parent-led behavioural interventions, alongside interventions that utilise various aspects of computer-based technology due to its intrinsic motivation for many of the autistic community (Autistica, 2016, Azad *et al.* 2018, 2021). No study to date has yet explored the feasibility of implementing a dance-based exergame in multiple contexts, i.e. the home and school environment, for autistic children. Therefore, the present study aims to explore the feasibility of *Just Dance* in both the school and home environments to overcome single-setting limitations.

## Aims and hypotheses

To date, there is no research specifically examining the feasibility and effectiveness of a dance-based exergaming protocol for autistic children’s social communication skills across multiple locations (i.e. home and school environments). Therefore, the overarching aim of the current research project is to assess the feasibility, guided by Bowen *et al.*'s (2009) criteria for feasibility studies, and the potential efficacy of *Just Dance* to enhance autistic children’s social communication skills in the home and school environment. To this end, we hypothesise that *Just Dance* will be a feasible intervention to implement in both environments (H1) and children will enjoy the intervention (H2). Furthermore, we hypothesise that participating in frequent sessions of *Just Dance* may elicit positive changes in the social-communicative skills of young autistic individuals, which will be apparent through the application and comparison of validated outcome measures and the analysis of behavioural observations within-subjects at pre-and post-intervention (H3).

## Methods

### Research design

Following a consultation with the autistic and wider autism community to discuss the proposed feasibility study design, the finalised research proposal was submitted for ethical approval. Outcomes from the consultation can be found online here: https://osf.io/kh86b. The proposal was pre-registered on the ‘As Predicted’ platform and the preregistration information can be found here: https://aspredicted.org/kc7p7.pdf. Ethical approval for the study was then given by the Science and Health Ethics Committee at the University of Essex (Ethics ID: ETH2122-0085).

A mixed-methods, within-subject repeated measures study design was employed to assess the feasibility and potential effectiveness of *Just Dance* in the home and school environment for enhancing autistic children’s social communication skills. Quantitative and qualitative outcome measures were completed pre-, during, and post-intervention by either parents (home-based study) or staff members who worked closely with each child at each school (school-based study).

### Participants

To employ voluntary response sampling, a range of recruitment techniques were utilised, similar to that of Fletcher-Watson *et al.* (2016) and Gao *et al.* (2019), to promote the feasibility study. These included word of mouth, generating a recruitment poster, and contacting schools, special education schools, and charities to share the study’s recruitment poster.

For the home-based feasibility study, eight eligible child-parent dyads met the inclusion criteria and consented to participate (Table 1). However, only five completed all of the pre-intervention outcome measures and only four embarked on the intervention and completed the post-outcome measures. Therefore, a total of four autistic children (M_age_ = 8.25 years, SD = 0.50) and their parents participated in the full study. Social demographics, experience with videogames, and familiarity with *Just Dance* differed for each participating child-parent dyad and are displayed in Tables 2–4.

**Table 1.** Inclusion criteria for participants enrolling onto the research project.

Inclusion criteria
Diagnosis of ASD confirmed by a professional
Child must be aged between 8 – 12 years old
Must have access to *Just Dance* either *via* a games console or online
Must have access to videoconferencing software (i.e. laptop, tablet, or phone with video camera)
Must be willing and able to commit to the duration of the research project

**Table 2.** Social demographics of children who consented and participated in the project.

	Child A	Child B	Child C	Child D
Age	8 years old	8 years old	9 years old	8 years old
Sex	Male	Female	Male	Male
Length of diagnosis	Over four years	Over four years	Over four years	Over four years
Additional diagnoses reported by parent	Dyslexia and Phonological Speech Disorder	None reported	None reported	None reported
School attended	Mainstream education	Mainstream education	Mainstream education	special education

**Table 3.** Social demographics of parents who consented and participated in the project with their child.

	Parent A	Parent B	Parent C	Parent D
Age	41–45 years old	41–45 years old	50+ years old	41–45 years old
Sex	Female	Female	Male	Male
Relation to child	Mother	Mother	Father	Father
Education	Level 7	Level 7	Level 2	Level 7
Occupation	Employed (Part-time)	Employed (Full-time)	Employed (Full-time)	Employed (Full-time)
Marital status	Married	Married	Married	Married

**Table 4.** Children’s videogaming experience and familiarity with Just Dance at home.

	Child A	Child B	Child C	Child D
Plays regular videogames (at least once a week)	Yes	Yes	Yes	No
Hours per week spent playing videogames	10+ Hours	Less than 1 h	4 – 7 h	Less than 1 h
Familiarity with *Just Dance*	Not familiar at all	Moderately familiar	Not familiar at all	Not familiar at all

For the school-based feasibility study, seven autistic children were recruited from School One (Manchester), including six males and one female. Twenty-four children were recruited from School Two (Essex), including 16 males and eight females. The mean age of the combined groups (*n* = 31) was 10.87 years old (SD = 1.61). All children had a confirmed diagnosis of ASD and attended one of the special education provider schools. Due to teachers completing the questionnaires, data were not collected regarding children’s videogaming experience or familiarity with *Just Dance* at home.

In each school setting, three staff members consented to participate in the project with regard to data collection. All staff members (*n* = 6) were female and were educated to at least a level six qualification or above. Staff members were between 26 and 45 years old. Staff experience ranged from less than a year in their current setting to over five years working at their current school.

### Exergame intervention

Following a recent systematic review on exergaming for autistic children (Morris *et al.* 2022) and the consultation held with members of the autism community, it was anticipated that a six-week exergaming programme would be sufficient to maintain the attention of young autistic individuals and potentially elicit beneficial effects. Therefore, child-parent dyads were requested to complete 12 *Just Dance* sessions (two sessions each week at a convenient time) across a six-week timeframe. Morris *et al.*’s (2022) systematic review also suggested that exergaming sessions with autistic children typically lasted between 20 to 30 min. With the relevant literature in mind, the duration of home-based sessions was discussed with the consultation panel and initial sessions of 25 min was proposed. Given the average length of songs on the *Just Dance* platform, this equated to 5 songs in each session. For concrete guidance, 60 songs (five songs in each session) were to be completed across the intervention. Parents were required to complete each session with their child at home, as the involvement of parents in interventions is observed to be advantageous (Althoff *et al.* 2019, Oono *et al.* 2013). As discussed prior, *Just Dance* was used as a vehicle to practice and engage in mirroring, interpersonal synchrony, and attend to rhythmical stimuli.

**Figure 1.**
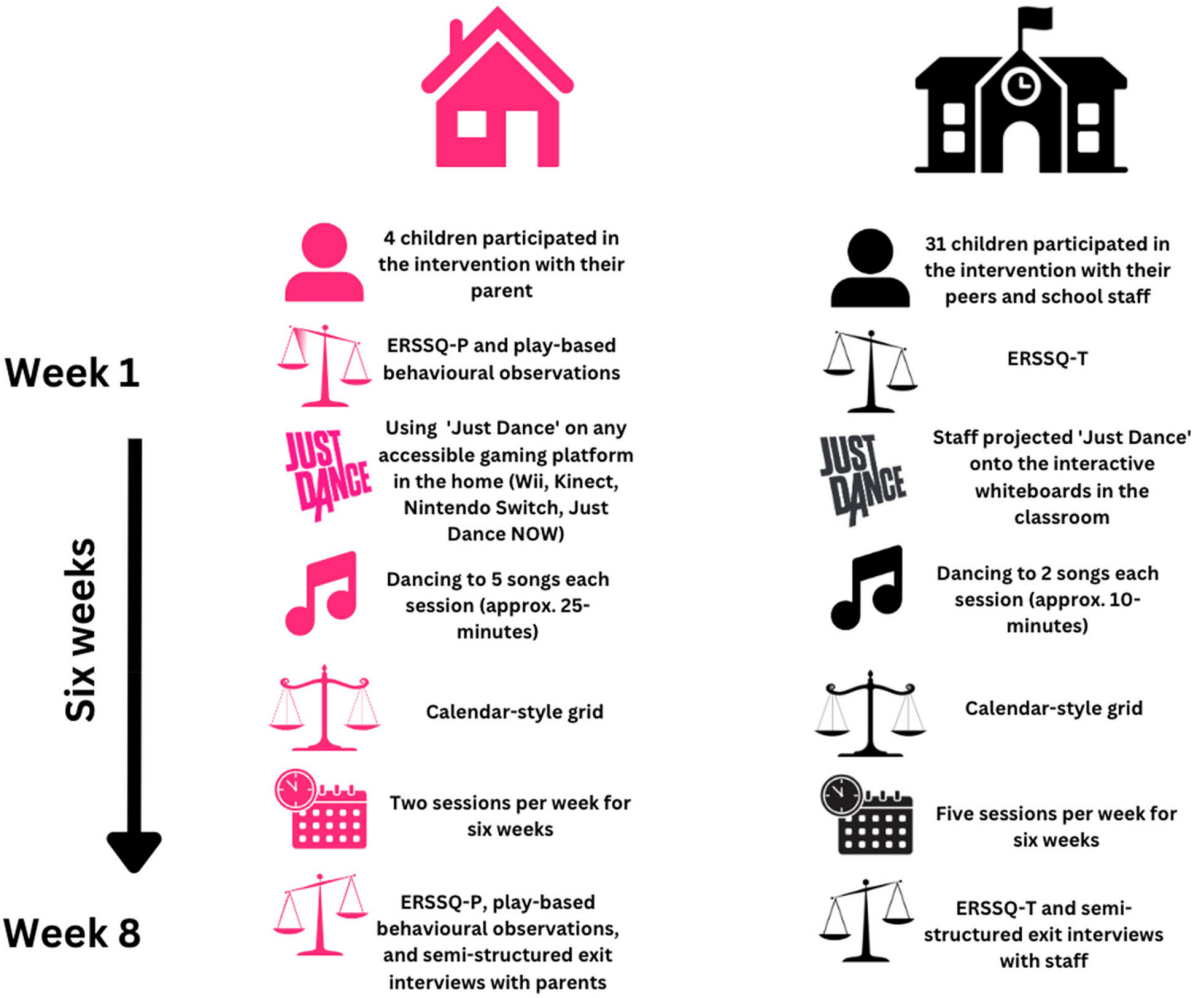
Schematic diagram representing the procedures for both the home-based study and the school-based study (Emotional Regulation and Social Skills Questionnaire Parent and Teacher version; ERSSQ-P/T, respectively).

Following suggestions from parents in the home-based study and consulting project liaisons from each school, 10-minute sessions were proposed for the school-based study instead of 25-minute sessions as employed in the home-based study. To ensure continuity in the duration of exergaming and interaction with *Just Dance* across the two locations (home and school), pupils and staff completed two songs every day on the *Just Dance* platform for one half-term (six weeks) in the school-based study. This equated to 60 songs, therefore children in the home-based study and school-based study were expected to spend the same length of time engaging with *Just Dance*, although on different schedules. It was suggested that staff members introduce the songs at the start of each school day. However, if pupils were not comfortable with these logistics, staff members were granted flexibility in their scheduling of the sessions to best reflect their pupils’ preferences. Staff projected *Just Dance* videos from YouTube onto the interactive classroom boards and asked children to follow the avatars on screen; joining in with the children and dancing across the six weeks. Staff were asked to give positive and motivating verbal feedback to children. An overall summary of the procedures for both studies is depicted in Figure 1.

### Measures

#### Adherence and enjoyability calendar

**Figure 2.**
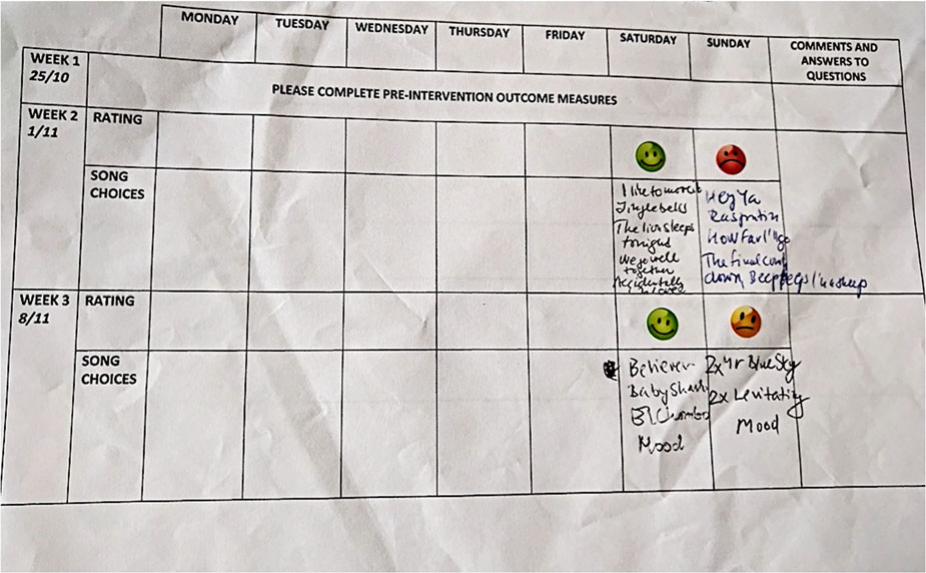
Photograph of the calendar-style grid and smiley face stickers that were sent to child-parent dyads at home and subsequently completed throughout the intervention to represent enjoyability of the session.

To help measure compliance and adherence to the intervention child-parent dyads were invited to mark each completed *Just Dance* session on a pre-distributed calendar-style grid (Figure 2). Children reacted to each completed session by choosing a sticker or drawing a face that best reflected their level of enjoyment based on a 3-point scale; similar to that found in the Fun ToolKit of Read and MacFarlane’s (2006) study. For example, using a green smiley face (2) for “I enjoyed the session”, using an orange neutral face (1) for “The session was okay”, and using a red sad face (0) for “I did not enjoy the session”. Parents were asked to specify what songs they completed each session and leave any comments for the week in a small text box.

Staff in the school-based study were also asked to complete the pre-distributed calendar-style grid, to help measure compliance and adherence to the intervention. Staff were expected to observe the children and offer an insight into the group’s level of enjoyment; rating the group’s perceived level of enjoyment from a 3-point Likert scale. A score of 2 indicated “most children enjoyed the session”, a score of 1 indicated “most children found the session okay”, and a score of 0 represented that “most children did not enjoy the session”. Parents and staff were asked to return the calendars to the research team at the end of the intervention.

#### Emotional regulation and social skills questionnaire (ERSSQ)

The Emotional Regulation and Social Skills Questionnaire parent version (ERSSQ-P; Beaumont and Sofronoff, 2008) was used to provide a standardised measure of the overall social performance of participants in the feasibility study. It consists of 27 items in the format of a five-point Likert Scale, from 0; never to 4; always. The questionnaire is designed to measure frequencies of effective engagement in social and emotional behaviours (e.g. “chooses appropriate solutions to social problems” or “deals effectively with bullying”). Further, the ERSSQ was developed in response to limitations of existing rating scales of social skills and was specifically created to measure behaviours that were targeted during various social skill interventions, rather than measure behaviours that differentiate autistic individuals from typically-developing individuals. It is, therefore, more likely to be sensitive to treatment effects (Butterworth *et al.* 2014). Beaumont and Sofronoff (2008) identified a single-factor structure of the ERSSQ. Therefore, a single total score is used as an indicator of social competence from both parent and teacher forms. Overall, the ERSSQ is observed to have good construct validity, with high internal consistency between the ERSSQ-P and ERSSQ-T. For example, the ERSSQ-P and the ERSSQ-T exhibited high internal consistency with Cronbach’s α coefficients of .90 and .92, respectively (Butterworth *et al.* 2014). The measure also converges with existing constructs in the expected direction and magnitude. For example, the ERSSQ-P shows a strong negative relationship with the social communication questionnaire (SCQ; Rutter *et al.* 2003) indicating criterion validity. Significant and positive correlations were also observed for parent and teacher forms with the social skills questionnaire (SSQ; Spence, 1995) suggesting good concurrent validity (Butterworth *et al.* 2014) Briefly, the SCQ is a reliable and recommended screening measure that was developed to identify traits associated with autism spectrum disorder. The SCQ consists of 40 items and a factor analysis of the SCQ supported a four-factor structure consisting of the following factors: social interaction, communication, abnormal language, and stereotyped behaviour (Snow, 2013). The SSQ focuses on social behaviours that are anticipated to influence the outcome of social interactions. The Social Skills Questionnaire includes 30 items, and like the ERSSQ, asks respondents to rate items which best describe the young person. It also has a single-factor structure (Spence, 2003). Several studies have used the ERSSQ since its development by Beaumont and Sofronoff (2008), including Tan *et al.* (2015) and Weiss *et al.* (2018).

For the school-based study, staff were asked to complete the teacher version of the same questionnaire (ERSSQ-T). The teacher version consists of 25 items, excluding two questions found in the ERSSQ-P that focus on behaviour in the home environment. The final section of the post-intervention ERSSQ-T included two additional questions designed to gain insight into children’s individual responses to the intervention and whether they enjoyed the experience across the six weeks. The first additional question asked staff members “Do you believe the child you support enjoyed the *Just Dance* sessions?”, was answered by a ‘Yes/Unsure/No’ response and the second question asked, “Please could you elaborate on your answer using the text box below”. Parents and staff completed the questionnaires *via* the Qualtrics platform one-week pre-intervention and one-week post-intervention.

#### Asynchronous behavioural observations (completed only in the home-based study)

To further assess the potential changes in children’s social skills in the home environment only, parents were invited to record 10-minutes of play with their child using an enjoyable activity one-week pre-intervention and one-week post-intervention. This activity was to be completed by the parent and their child only. Previous studies; including, Narzisi (2020) and Wainer and Ingersoll (2015) have used similar methods for observing the social skills of children. Asynchronous behavioural observations were not conducted in the school-based environment due to ethical restrictions raised by the data governance officers in each school setting.

Once recorded, the videos were forwarded to the research team for subsequent behavioural coding using the BORIS coding software (Friard and Gamba, 2016). Based on previous studies (Heimann *et al.* 2006, Nadel *et al.* 2000) a coding scheme and ethnogram were devised to measure behaviours reflecting social interest during the play-based scenarios. Coded behaviours were grouped into two broad categories; distal social behaviours and proximal social behaviours (Nadel *et al.* 2000; Table 5). The percentage of time each child spent displaying proximal and distal social behaviours was then used to provide an overall percentage spent displaying social interest during the pre-intervention and post-intervention play-based activities. All videos were coded by the first author and a blinded independent researcher (SMG), who was trained in the use of BORIS and was unaware of the time-points of the video recordings and the first author’s coded results. Inter-rater agreement was then calculated using Kappa’s coefficient to ensure the reliability of the coding scheme (McHugh, 2012). Reliability for the proximal and distal codes used in this study for each video achieved a kappa coefficient (*K*) greater than 0.86; suggesting near-perfect agreement between observers (Landis and Koch, 1977).

**Table 5.** Coding scheme used to code relevant behaviours during play-based observations.

Behaviour coded	Behaviour type	Description of behaviour	Behavioural category
Touching the adult	State event	Child makes physical contact with parent; hugging, hand holding, patting, holding arm, etc	Proximal Behaviours
Verbal interaction	State event	Child engages verbally with the parent; speaks to parent, makes vocalisations towards parents, (speech need not be words)	Proximal Behaviours
Physical interaction	State event	Child physically interacts with the parent; passes toy/item, shows parent toy/item	Proximal Behaviours
Eye contact	State event	Child makes eye contact with parent; eye contact is established between parent and child	Distal Behaviours
Look/Gaze	State event	Child looks or gazes at parent; looks towards or in the direct of parent or looks where parent has directed their attention	Distal Behaviours
Facial expression	State event	Child displays facial expressions; child’s facial expression differs from their neutral expression (can be positive or negative)	Distal Behaviours

To measure positive social affect and enjoyment during the *Just Dance* game, we invited parents to record the first, middle, and final *Just Dance* sessions of the intervention with their children and forward these recordings to the research team for behavioural coding. Behaviours established in the Dyadic Parent-Child Interaction Coding System (DPICS; Eyberg *et al.* 2005), specifically focusing on the duration of child positive verbal affect, child positive nonverbal affect, and child physical warmth were coded during these *Just Dance* sessions (Table 6). Codes were combined to form the percentage of time each child spent showing affect and enjoyment during the recorded sessions.

**Table 6.** Coding scheme used to code relevant behaviours during intervention-based observations.

Behaviour coded	Behaviour type	Description of behaviour	Behavioural category
Child positive affect verbal	State event	Any positive evaluative verbal expression of pleasure, warmth, enthusiasm, or gratitude such as “This is fun”, “Oh goody”, “I like playing these games”, “Thank you”, “I’m a winner” or singing along to the songs. However, words do not always need to be spoken to code this – any verbal positive expression.	Affect and Enjoyment
Child positive affect nonverbal	State event	Nonverbal expression of enjoyment, warmth or enthusiasm directed at the parent or game, such as smiling and laughing, or directing positive gestures towards the parents/game	Affect and Enjoyment
Child physical warmth	State event	Child physical warmth is an explicit physical act of endearment initiated by the child or positively received by the child (positive physical interaction); such as hugging, touching the adult, climbing onto the adult, high-five, patting the parent on the body	Affect and Enjoyment

To establish reliability, 25% of the *Just Dance* videos were selected at random and coded by the first author and a blinded member of the research team (EH), who was also trained in the use of BORIS and was unaware of the time-points of the video recordings and the first author’s coded results. Inter-rater agreement was calculated using Kappa’s coefficient to ensure the reliability of the coding scheme (McHugh, 2012). Reliability for the intervention-based observational codes used in this study was above *K* = 0.80 for 25% of the randomly selected intervention-based videos. The first author then continued to code the remaining 75% of the *Just Dance* videos once near-perfect agreement had been established (Landis and Koch, 1977).

#### Semi-structured interviews

During the home-based intervention, one informal telephone conversation with each parent in the third week was hosted to assess how the intervention was going and to mitigate any potential barriers for the remaining weeks. A follow-up document was then sent to parents with helpful suggestions to overcome any barriers that were apparent (see https://osf.io/anqp6)

At the end of the intervention, a 30-minute semi-structured exit interview was held with parents and staff *via* Zoom videoconferencing to discuss the overall feasibility of the intervention and discuss children’s enjoyability. Questions focused on the recruitment and training phases, intervention, use of outcome measures, and suggestions for future projects for the home-based study (Table 7). For the school-based study, topics such as the ‘recruitment phase’ and ‘training phase’ were not included and instead focused on the ‘planning phase’. Both semi-structured interviews focused on the context (school or home) in which the intervention was delivered.

**Table 7.** Topics of semi-structured interview and leading questions asked to structure the interview with parents.

Topic	Leading question asked
Recruitment phase	How did you find the recruitment phase?
Training phase	How did you find the training phase of the project; this includes both the training webinar and training manual/handbook?
Feasibility of intervention	How did you find the intervention overall; i.e. the six-week *Just Dance* programme?
Feasibility of outcome measures	How easy did you find it to complete all the outcome measures, including the parent rating scale, in-home recording, and calendar style grid?
Enjoyability and effectiveness *via* parent recall	Do you think your child enjoyed participating in the project and do you think it has the potential to enhance social communication skills?
Potential improvements for future projects	How did you find the project from start to finish, and do you have any suggestions on how to improve the project?

### Data analysis

The quantitative data (ERSSQ-P/T responses and coded behavioural observations) were analysed using SPSS (*IBM SPSS Statistics*, 2017) and GraphPad Prism 9 (*GraphPad Software*, 2020); where analyses were carried out, frequency tables were produced, and graphs were created. To establish whether data were suitable for parametric testing, Shapiro-Wilk tests were employed due to the small sample size. Once normality had been established, paired and parametric t-tests were carried out on the datasets to assess whether the play-based behavioural observations and ERSSQ (both parent and teacher) were capable of detecting variances that might infer a change in social-communication skills from pre- to post-intervention. Confidence levels (Cl) were calculated around the mean difference and effect sizes (represented by Cohen’s d (d)) were also determined by dividing by the pooled standard deviation (Lakens, 2013).

Qualitative analyses were conducted in order to establish the feasibility of introducing an exergaming intervention into the home and school environment from the perspective of both parents and school staff. Thematic analysis, using the NVivo12 software (*NVivo*, 2018), was carried out on the transcribed semi-structured interviews. Briefly, thematic analysis is a flexible qualitative method that allows for the identification, analysis, and reporting of patterns or themes within data (McLemore *et al.* 2014). Where themes capture important elements of the data in relation to the research question and represent some level of patterned response or meaning within the datasets (Braun and Clarke, 2006, Swart, 2019). To this end, the interview transcripts were systematically read several times by the lead author to ensure familiarity with the dataset. Words, phrases, and sentences were categorised under different codes until code saturation was perceived (but ultimately never confirmed) by the first author (Braun and Clarke, 2021). Initial themes and sub-themes were generated by clustering similar codes together. These themes were then discussed and reviewed with the research team. During this discussion, domain summary themes; themes that “aim to capture the diversity of meaning in relation to a topic or area of focus” (pg. 593; Braun and Clarke, 2019), were deemed most appropriate to conceptualise themes and create a narrative. Thus, presenting an extension of Braun and Clarke’s (2006) thematic analysis to provide a narrative more suited to this report.

## Results

### Adherence to the intervention and enjoyment

Only three child-parent dyads (A, B, and C) returned their calendar-style grids from which to measure their adherence. A verbal discussion was held with parent D to retrieve an approximation of the necessary information that would have been on their calendar-style grid. On average, the group completed 61% of the sessions; approximately seven sessions out of 12. The most common number of songs completed each session was three and the most common rating for the sessions was 2 – “I enjoyed the session”. A range of songs was chosen for each session by child-parent dyads; however, most of the songs selected had a 4/4 time signature, moderato-allegro tempo, and included age-appropriate pop lyrics. These included Katy Perry’s ‘Roar’, Pharrell’s ‘Happy’, and Shakira’s ‘Waka Waka’.

School One (Manchester) reported that they completed 100% of the sessions; engaging with two *Just Dance* videos each day. They reported varying levels of participation from the children, however, on average 71% of the children participated in the sessions each day. School Two (Essex) reported that they completed 90% of the sessions; engaging with two *Just Dance* videos each day. They reported varying levels of participation from the children, however, on average more than 75% of the children participated in the sessions each day.

With regards to children’s enjoyability in the school environment, sessions were typically rated as 2 – “children enjoyed the session” on the calendars from both schools. Furthermore, at the end of the six-week intervention, staff from each school reported that more children enjoyed the sessions than those that did not or those that they were unsure about. Specifically, 20 children were reported to enjoy participating in the intervention, whereas 3 children were reported to not enjoy participating in the intervention, and 7 children showed varying levels of enjoyment during the intervention meaning staff were unsure of the extent to which they enjoyed the experience.

Staff feedback from the additional text box answers at the end of the post-intervention ERSSQ-T reflected on children’s individual experiences of the sessions. Most reported that children “looked forward to the daily *Just Dance* sessions” and “enjoyed dancing”. One child was said to be “up at the front and leading the class” and another child “loved dancing and participated very well – you could see how much he enjoyed copying the moves”. However, staff reported that some children “did not join in at all” or were “shy” and needed “a little encouragement”. For example, one staff member stated “to begin with [one child] did not want to participate but he soon gained in confidence and participated more towards the end of term”.

### Emotional regulation and social skills questionnaire

**Figure 3.**
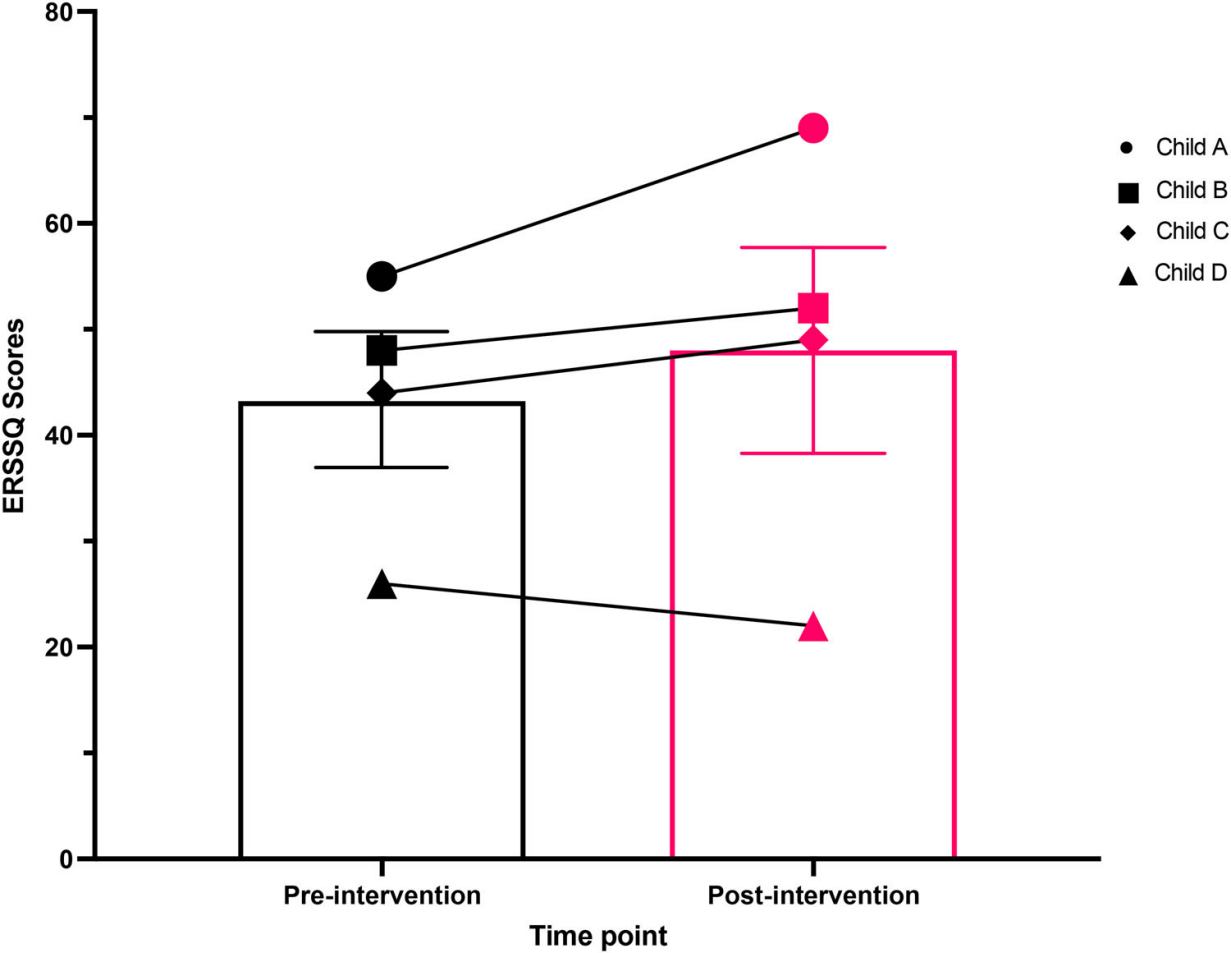
Mean (±SEM) bar chart overlaid by an individual stacked scatterplot of children’s (*n* = 4) Emotional Regulation and Social Skills (ERSSQ) scores before and after six weeks of Just Dance sessions at home. Key shown on graph for each child’s individual dataset marker.

The group means and children’s individual scores from the home environment for the pre-intervention and post-intervention ERSSQ-Ps are displayed in Figure 3 . Results from the two-tailed, paired, and parametric t-test on the ERSSQ-P data revealed a statistically non-significant small-medium positive effect between pre-intervention and post-intervention, (*n* = 4, t(3) = 1.290, *p* = 0.287, 95% CI [-6.970, 16.470], Cohen’s *d* = 0.291). The lack of statistical significance was most likely due to the small sample size. On average, the group’s ERSSQ-P score improved by 4 points from pre-test to post-test.

**Figure 4.**
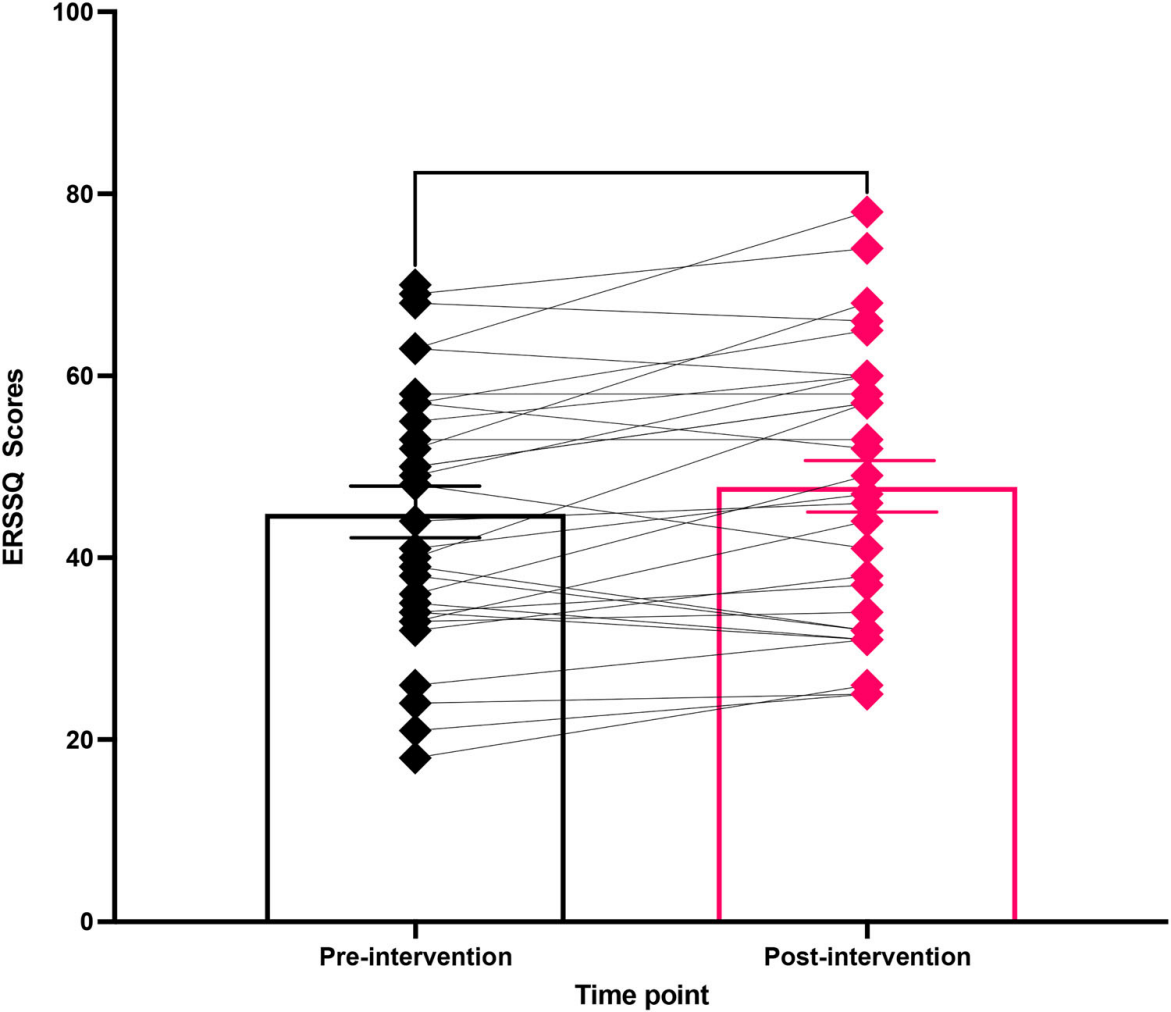
Mean (±SEM) bar chart overlaid by an individual stacked scatterplot of children’s (*n* = 31) Emotional Regulation and Social Skills (ERSSQ) scores before and after six weeks of Just Dance sessions at school. A two-tailed, paired, and parametric t-test revealed a significant difference between pre- and post-intervention means for the group; *p* < 0.01 (**).

The combined group means for participating children in the school environment for the pre-intervention and post-intervention ERSSQ-T are displayed in Figure 4 . Results from the two-tailed, paired, and parametric t-test on the ERSSQ-T data revealed a statistically significant improvement in children’s ERSSQ-T scores between pre- and post-intervention (*n* = 31, t(29) = 3.052, *p* = 0.005, 95% CI [2.167, 5.433], Cohen’s *d* = 0.200). On average children’s scores improved by 4 points on the ERSSQ-T from pre-test to post-test.

### Play-based observations

**Figure 5.**
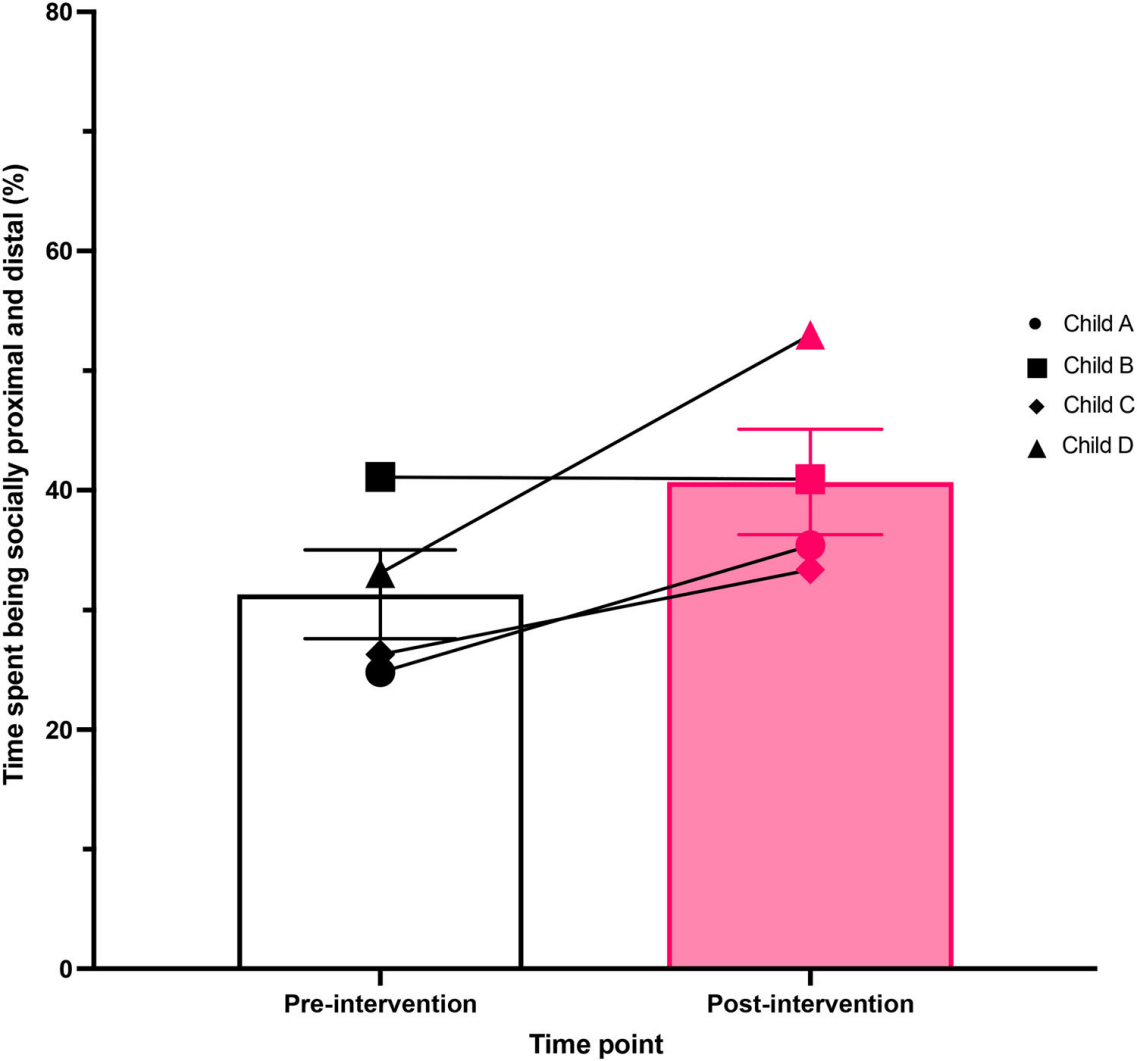
Mean (±SEM) bar chart overlaid by an individual stacked scatterplot of the percentage of time children (*n* = 4) spent showing social interest (displaying proximal and distal social behaviours) towards the adult during the play-based observation recorded before and after six weeks of Just Dance sessions. Key shown on graph for each child’s individual dataset marker.

Each child completed an enjoyable activity for 10-minutes with their parent. These activities were videorecorded and subsequently coded by the research team. Child A played with train and plane models, Child B baked and decorated cakes, Child C played chess, and Child D completed word puzzles. The group means and children’s individual percentages for time spent displaying socially proximal (touching the adult, engaging with the adult physically, and verbal interactions) and socially distal (initiating or maintaining eye contact, showing joint attention *via* gaze or looking, and displaying facial expressions) behaviours towards their parent are presented in Figure 5. Results from the two-tailed paired t-test on the play-based behavioural observation data revealed a large improvement between pre-intervention and post-intervention (*n* = 4, t(3) = 2.240, *p* = 0.111, 95% CI [-3.936, 22.640], Cohen’s *d* = 1.146). Although a large effect was observed, this was deemed statistically non-significant - most likely due to the small sample size in the home-based feasibility study. Three out of the four children showed a positive increase in overall social interest (determined by the percentage of time spent being socially proximal and distal) during their play-based observations between pre- and post-intervention. Child B remained the same on this measure.

### Intervention-based observations

**Figure 6.**
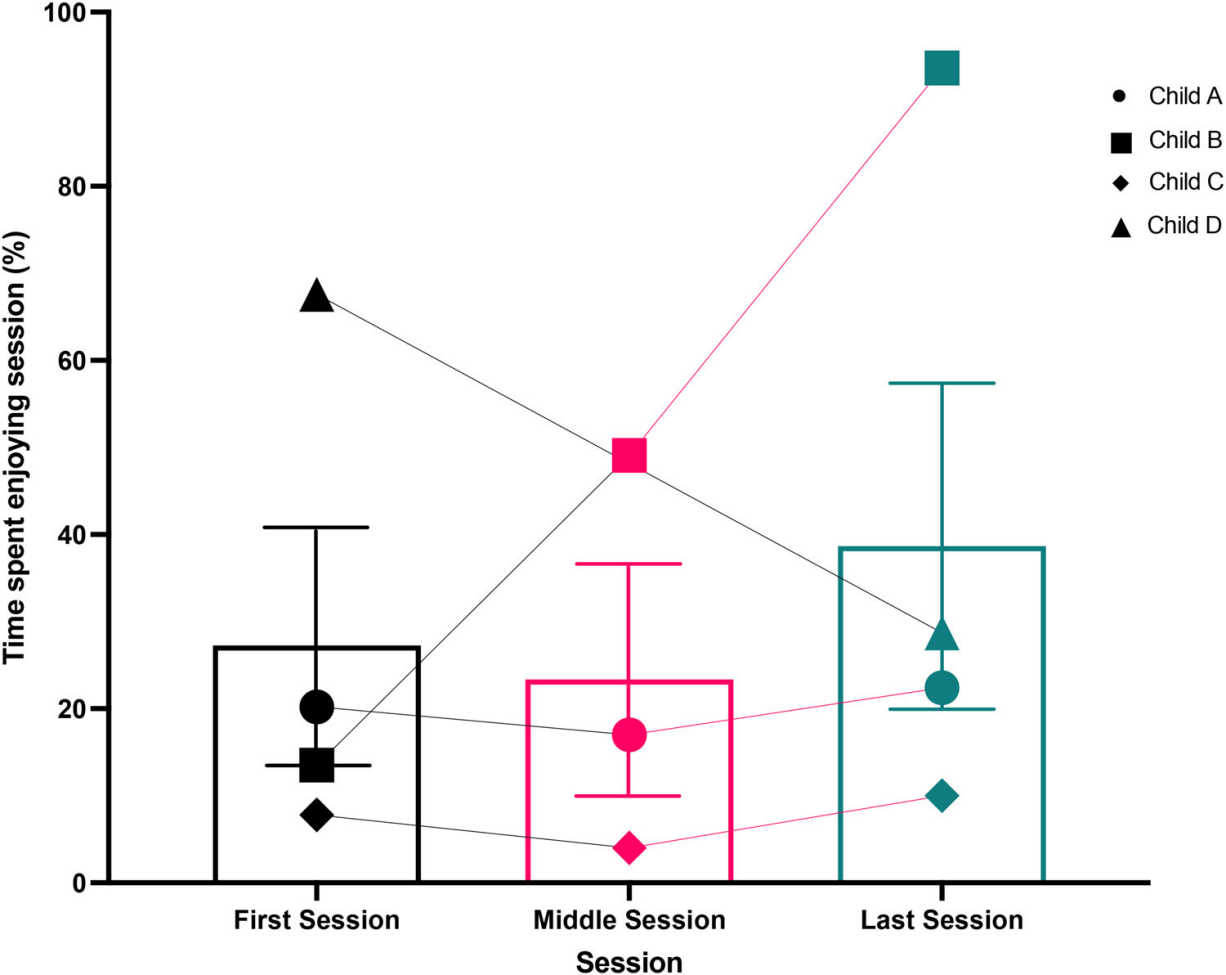
Mean (±SEM) and individual percentage of time children (*n* = 4) spent enjoying the session (displaying positive verbal and non-verbal affect and physical warmth towards the adult) during the first, middle, and last recorded Just Dance sessions. Key shown on graph for each child’s individual dataset marker.

The group means and children’s individual percentages for perceived time spent enjoying the first, middle, and final *Just Dance* sessions are presented in Figure 6. Parent-child dyad D were unable to return their middle session recording. Children were observed to either maintain or increase their levels of enjoyment across the recorded sessions; as demonstrated by verbal and nonverbal positive affect and physical warmth directed to or received by the adult.

### Thematic analysis of transcribed exit interviews

Seven key domain summaries were identified following thematic analysis of the transcribed semi-structured exit interviews with parents in the home-based study. Each domain summary included a variety of related key themes, sub-themes, and nodes. A detailed overview of the key domain summaries, themes, sub-themes, and specific codes with quote examples can be found in the supplementary material (Table SM1). The order of domains reflects the chronological order of the study, beginning with the organisation of the project as perceived by the interviewees and then the importance of providing children with choice - something that has not been well-document in the research before. Domains are then presented relating the feasibility of the project, positives, challenges, and improvements for future studies – an important domain to finish on to help inform future work (Figure 6).

**Domain summary one:** Appreciation for the organisation and the information provided throughout the project.

Parents were forthcoming in highlighting the successful organisation of the project and expressing their appreciation of the information provided. Thematic analysis of the transcripts indicated that parents valued the taster and training webinars, as they provided a “build-up” to the project and ensured relevant information was conveyed. Parents also enjoyed the hybrid training approach, where an online training webinar was held and an electronic training manual was distributed. Parents were keen to convey that the organisation of the project was “straightforward”, “easy to navigate”, and “streamlined”, expressing appreciation for the visual presentations, design of the materials that were made available, and clear instructions. Furthermore, parents valued being able to contact a member of the research team either by WhatsApp or email at a time that was convenient to them.

**Domain summary two:** Importance of preparing children and offering them options before and during the intervention.

The second domain summary identified was the importance of preparing children for the introduction of the intervention and the significance of “enabling a choice” in order for each child to feel more “in control” of their intervention. Many parents informed that giving their children “options” and allowing them to choose was “comforting” and ensured that they were more “willing to take part”. This was facilitated by giving children the option to choose what songs they wished to complete each session. Parents also suggested preparing their children for the intervention was important. Therefore, parent B showed her daughter the recorded webinars to help her understand why they were starting *Just Dance* and preselected the songs with her daughter before each *Just Dance* session.

**Domain summary three:** Personal circumstances affecting the delivery of the intervention.

As the intervention was delivered at home, analysis of the transcripts emphasised differing family-specific circumstances that affected the delivery of the intervention. There were various personal commitments that each family encountered, which restricted the number of sessions they completed. For example, one child-parent dyad was on holiday the first week of the project and another family fell ill with a cold during the intervention. Yet, each parent felt that two sessions a week were “do-able”. Furthermore, parents spoke about the importance of “creating structure and routine” in their home and that, for at least two parents, “doing it on a weekend” helped to achieve this structured routine.

**Domain summary four:** Feasibility of outcome measures used within the project.

Several parents informed that the outcome measures were “easy to use” and complete, including the ERSSQ-P, play-based recordings, and the calendar-style grid. However, one parent felt that the ERSSQ-P could be made more “individualised” to reflect the skill set of their child. Additionally, parent D reported losing their calendar-style grid halfway through the intervention, suggesting a possible downfall of the feasibility of the calendar-style grid. Nonetheless, parents appreciated the visual aspect of the calendar-style grid and the use of different coloured faces to reflect levels of enjoyment.

**Domain summary five:** Positives to take away from the project.

Overall, parents provided positive feedback and expressed that they would be “happy to continue” with the intervention at home. Furthermore, parents emphasised the importance of the intervention being “physically active” and being able to use dance as a way of “expression”. Parents were keen to participate in the project as they could see “value” in movement aiding communication skills, were keen to “learn something”, and have “an enjoyable experience” with their child. Importantly, parents found using *Just Dance* “easy and not too onerous” and appreciated that it could be used on various platforms, including Nintendo Switch, XBOX Kinect, and also YouTube. Again, providing options for children to choose what platform they would like to complete the sessions on. Parents also expressed that their children enjoyed playing *Just Dance*, especially the “visual aspect” and being able to “follow” the avatars on screen. Many parents appreciated that this involved children “mirroring” the avatar and moving in synchrony with one another. All parents explained that their children had an innate “love for dancing and music” and were able to express themselves through the non-verbal realm of movement, indicating the prosperous use of dance-based exergames.

**Domain summary six:** Challenges to the intervention.

Despite the many positives highlighted by parents, some challenges to the intervention were raised. Parents expressed concerns that their children were “burned out” and that introducing another activity to their schedules may “overload” them. Furthermore, parents informed that children often associated “home-time with downtime” and, therefore, were occasionally reluctant to take part in the intervention – suggesting a drawback of home-based interventions. A negative aspect of utilising the *Just Dance* platform was that sometimes dances were “too fast” for children to follow accurately, making the experience “challenging”.

**Domain summary seven:** Potential improvements and changes for future studies.

Parents suggested various changes that could be made to the protocol to improve it for future studies. For example, “providing a more in-depth social story” to explain the intervention. Interestingly, all parents suggested five songs were “too much” for their child. As a result, many child-parent dyads completed two or three songs together instead. This was also reflected in their calendar-style grids. Furthermore, one parent echoed that some of the songs available on the *Just Dance* platform were “too fast” and proposed that the videos could be made “slower so that they are easier to follow”. Parents also suggested providing an “incentive”, such as a “five-pound voucher” to improve children’s “buy-in” to the project.

Similar to the home-based study, six key domain summary themes were generated during the thematic analysis of the transcribed semi-structured exit interviews with staff members in the school-based study. A detailed summary of the outcomes with verbatim comments can be found in the supplementary material (Table SM2). A similar chronological order for the presentation of domains was employed as above in the home-based study.

**Domain summary one:** Appreciation of collaborative planning, organisation, and information provided throughout the project.

Staff indicated that sufficient information was provided throughout the intervention and that they felt well-informed about what was required to complete the project. Staff suggested that they felt “prepared to deliver the intervention” and key staff members were able to “train” other participating colleagues, such as assistants and support workers, to deliver the intervention in the school environment. Interviewed staff members also highlighted the responsiveness of the research team and valued their “quick responses” to resolve any issues or answer any questions. A key outcome of the successful planning and organisation was that staff members felt able to participate in the intervention with children and “support pupils to take part”.

**Domain summary two:** Importance of creating structure and routine both within the sessions and throughout the intervention.

Staff were keen to ensure that the intervention could be “built” into the children’s routine and felt that completing the intervention daily made this easier. Furthermore, this approach helped to manage children’s anxieties as they began to “expect” the intervention day-to-day. Staff were also able to create structure within each session by using the songs and their differing tempos; for example, starting with a high-energy dance and song, then finishing with a steady-energy dance and song to signify the session was “drawing to a close”.

**Domain summary three:** Emphasising the individual-ness of each child within the classroom.

Interviewed staff members were eager to emphasise that all pupils were different and would respond differently and participate in different ways to one another. For example, some pupils “danced with confidence at the front of the classroom”, whilst other pupils danced near their support workers and other pupils simply “watched the other children dancing in the classroom”. The exergaming intervention permitted this “individual participation”. Furthermore, staff were keen to integrate the intervention into the children’s school day at a time that was most beneficial for children; for example, using the intervention to help “pick up the children” if there was an energy lull or to “take a break in between maths” and “re-energise”.

**Domain summary four:** Benefits of participating in the intervention and positives of the project, including ease of implementation and flexibility.

Domain summary four was the largest summary containing many themes and subthemes that covered a vast array of comments made by staff members focusing on the benefits of the intervention. These included its ease of implementation and flexibility, alongside providing a vehicle to “bring children together” and engage in “music and movement”. Staff suggested that adherence to the intervention was relatively easy to achieve and agreed that two songs were an appropriate length of time to maintain children’s attention and enjoyment. Staff highlighted the flexibility of the exergaming intervention and being able to fit it into the children’s schedules were key benefits of the intervention itself and the project. Similarly, the flexibility of being able to complete the intervention in different environments; for example, inside the classroom or outside the classroom, based on the child’s preference further, enabled staff to cater to different children’s requirements. Furthermore, the variety of songs and dances available meant that pupils and teachers could be flexible and find the most appropriate and liked songs for each class, enhancing enjoyability and participation.

Staff emphasised the positives of the intervention for children and stressed the “importance of physical activity” and movement for their pupils, alongside children “enjoying music” and using the intervention as an outlet to help “regulate” mood and emotions. Staff also suggested that they could see the “potential of *Just Dance* to support learning” and “help with [children’s] attention” during class. Additional positive outcomes that can be taken away from the project include staff continuing with the intervention following completion of the study, their willingness to “recommend the intervention to other schools and classes”, and ease of completing the outcome measures. Furthermore, an encouraging subtheme generated in relation to the questionnaire was the school’s desire to “increase awareness” of ASD in academic research. Specifically, the school wanted to highlight that often their pupils are “complex” and a ‘one size fits all approach’ is not appropriate with regards to the questionnaire used within this feasibility study, raising the issue is “there a better way of doing this” and reaffirming the need for person-centred research and outcome measures.

**Domain summary five:** Challenges to the intervention and drawbacks of the project.

Domain summary five encompasses challenges to the project, including the negatives of the questionnaire and the reliance of staff to integrate a new activity into the school day, who were already “stretched”. It also highlights drawbacks to the intervention, such as the songs being “too fast” or potentially overwhelming for some children. Although staff found the outcome measures easy to complete, one school highlighted that items on the questionnaire did not always “relate to skills of the children when taken at face value”. In the thematic analysis, it was identified that a more “abstract view” of specific items on the questionnaire may help to mitigate this challenge.

**Domain summary six:** Adaptations to the protocol during delivery and suggestions for future studies.

The final domain summary focuses on adaptations to the protocol staff members implemented during the intervention and suggestions to improve its application and uptake. One staff member introduced a “*Just Dance* Champion” to enhance children’s motivation, whereby pupils engaging and participating in the intervention were able to choose the next song. This enabled many pupils to enjoy the “freedom of choosing different songs” and have “ownership” over the sessions. Staff members felt that they soon became “facilitators” of the sessions, allowing children to choose the songs they wanted to complete and “leading the sessions” themselves. Another staff member found that “splitting” children up into groups based on their needs and preferences during the sessions was beneficial, rather than keeping all children in the classroom at one time. Suggestions for future studies included creating visual instructions to introduce the sessions and selecting staff members who had the greatest capacity to complete the outcome measures, specifically the ERSSQ-T. Another interviewed staff member proposed the idea of working with other groups of pupils, including “older pupils” and pupils with additional needs outside the realms of autism as they believed that the intervention could hold beneficial effects for different groups of pupils and adolescents.

## Discussion

The present studies assessed the feasibility and potential effectiveness of a dance-based exergame in both the home and school environments for enhancing autistic children’s social communications skills. It was hypothesised that the intervention would be feasible to implement in both the home and school environment (H1) and that children would enjoy the intervention (H2). The results suggested that most children found the exergame enjoyable, the intervention could be tailored to each child, and parents/teachers were able to implement *Just Dance* sessions into their respective environments; applying minor adaptations to the protocol to improve its feasibility and uptake. Semi-structured interviews afforded high-quality, in-depth, and context-specific discussions regarding the feasibility of the current protocol from both the parent’s and school staff members’ perspectives. Importantly, for a feasibility study, no adverse outcomes were reported from parents/teachers or children participating in the project, and all children who started the intervention in week one continued to week six, indicating there were no ‘drop-outs’ from the intervention.

It was also hypothesised that participating in frequent *Just Dance* sessions may elicit positive changes in the social-communicative skills of young autistic individuals (H3). The results from both the home- and school-based studies suggest a small-to-medium positive effect in children’s ERSSQ scores and/or behavioural observation scores at home and a statistically significant improvement in children’s ERSSQ between pre-test and post-test for the school-based study. On average both studies demonstrated a four-point improvement on the ERSSQ outcome measure.

### Social and educational implications

As addressed in the aims, the purpose of this study was to assess the feasibility of the intervention guided by Bowen *et al.* (2009) criteria for feasibility studies. Overall, the results described above indicate that the intervention is acceptable, holds demand, can be implemented into the home and school environments, allows for adaption and integration, and with minor adaptions to the scheduling in the home environment is practical for children. For example, results from the present feasibility studies suggest that *Just Dance* is an acceptable intervention as it was enjoyed by most autistic children in the two studies. This is similar to previous research indicating that autistic children enjoy and prefer dance-based exergames (Morris *et al.* 2022). Similarly, the intervention was able to be implemented into the respective environments with ease. Parents and staff did not report a great level of adaptation or change needed within their environments to implement *Just Dance*. Thematic analysis of the transcribed interviews suggested parents and staff were willing to continue with the intervention after the project had finished suggestive of demand for the intervention. This was also supported by no dropouts. Previous research has indicated that interventions utilising mirroring and rhythms techniques also often have low drop-out rates - similar to the results of this feasibility study (Heimann *et al.* 2006, Katagiri *et al.* 2010, Reese, 2018, Willemin *et al.* 2018). As parents were able to use videogaming platforms that they and their children already had access to and the schools used freely available videos on YouTube to access *Just Dance*, the cost of the intervention was minimal and did not impact the participants financially, indicating successful practicality of the intervention, Therefore, the importance of evidence-based and freely accessible interventions for autistic children is highlighted. On average parents and children completed 61% of the 12 sessions and two-to-three songs each session. Whereas in the school-based study, children and staff completed over 90% of sessions completing two songs each session. As a result, the integration of the intervention appears more feasible in the school-based environment and children seem more amenable to completing two songs each session. To our knowledge, this work is the first (and only) research paper to explore the feasibility of a dance-based exergame for enhancing autistic children’s social communication skills in multiple locations. It emphasises the need for researchers to ensure they are conducting and publishing feasibility studies to confirm the acceptability of an intervention, how well it integrates into an environment of choice, and if any adaptations are necessary before conducting larger efficacy trials.

These preliminary findings suggest that participating in short and frequent *Just Dance* sessions may positively influence autistic children’s social communication skills. The successful inclusion of mirroring and rhythm within the exergame may have contributed to these advances in perceived social communication skills and emotional regulation. Furthermore, *Just Dance* provided a vehicle for children to engage in a physical activity together with their peers at school or with their parents at home; encouraging interpersonal motor synchrony. Despite a small minority of children preferring to complete the sessions in smaller groups or 1:1 with their support workers at school, many children remained in the classroom and participated in the sessions together. Thus, facilitating an opportunity for communication and social interaction between peers and child-parent dyads and encouraging interpersonal synchrony due to completing similar movements at the same time. Importantly, a multitude of studies have indicated that increased interpersonal motor synchrony (Cirelli *et al.* 2014, McNaughton and Redcay, 2020, Rennung and Göritz, 2016) and improved imitation skills (Cardon and Wilcox, 2011, Field, 2017, Ingersoll, 2012, Wainer and Ingersoll, 2015) have been associated with an increase in prosocial behaviours, empathy, connection between social partners, and social development.

The results from this feasibility study indicate that *Just Dance* is an enjoyable physical activity for parents, teachers, and children to complete. Notably, the multi-environment approach suggests that *Just Dance* could be used as a low-cost medium to support physical activity engagement and potentially aid social communication skills, whilst being accessible to geographically dispersed participants in multiple settings and not relying on continuous input from researchers or the attendance of a trained professional to implement it. Despite, all participants completing the same activity, the exergame afforded a flexible approach that could be tailored to each child’s desires; what songs they would like to complete, the environment they would like to complete it in, how many songs they would like to do, and the time they would like to complete it. Such factors present the opportunity for children to have ownership over their intervention; enabling a person-centred approach whilst completing the same activity. Moreover, children can partake in the intervention both at home and school; overcoming the challenges of single-setting interventions and enabling children to become more aware of the exergame and its expectations, leading to greater familiarity and a sense of comfort within the exergame (Bernardini *et al.* 2014, O’Connor *et al.* 2009).

### Limitations

Despite the many encouraging outcomes that can be observed from these dual small-scale feasibility studies; including the delivery of the intervention in two separate specialist education provider locations, in the home environment, and the positive reception of the intervention by parents, staff, and pupils, the present study is not without its limitations. The small sample size and absence of an active control group only permitted limited-efficacy testing, subsequently constraining null hypothesis statistical testing. As a result, it is difficult to ascertain the true effectiveness of the dance-based exergame on children’s social communication skills and the generalisability of the results and their statistical significance is somewhat restricted. Similarly, the qualitative data and experiences of parents and teachers were specific to each of their respective environments. Therefore, the findings are context-specific and from a relatively small sample. Nonetheless, the ease of implementation was consistently reported across both settings. Similarly, the increase in reported social skills found in most individuals was consistent across both feasibility studies, with a similar four-point improvement in ERSSQ scores. Moreover, observational data from the home-based study enabled some confirmation of enhanced social skills between pre- and post-intervention for the group.

The measure of children’s social communication skills was predominately based on one informant from each respective location (i.e. school – teachers and home – parents), which could introduce a rating bias. Butterworth *et al.* (2014) recommend using a variety of measures, including both teacher and parent measures in a single study, to obtain a comprehensive assessment of children’s social communication skills and understand the true effectiveness of an intervention. It was highlighted that some parents and staff felt a more “abstract view” of specific items on the chosen questionnaire was needed to truly reflect the skills of some children. Therefore, the inclusion of additional outcome measures, which have been discussed with the autistic and wider autism communities and focus on autism-prioritised outcomes is necessary. For example, the International Consortium for Health Outcomes Measurement (ICHOM) recently produced a standardised ‘Autism Spectrum Disorder Standard Set’ (ASDSS) of recommended outcome measures based on the input from leading autism researchers, psychologists, board-certified behaviour analysts, and, most importantly, autistic representatives from Europe, North and South America, and Asia (Frechter *et al.* 2022, ICHOM, 2021).

Whilst providing a naturalistic insight into children’s social-communication processes and enjoyment with their parent during the home-based study, the observational outcome measures relied on human observations to code the play-based and intervention-based recordings into meaningful and usable data. Subsequently, biases and human error may have been introduced. Although steps were taken to minimise the introduction of bias (i.e. multiple and blinded raters), software such as OpenPose (Cao *et al.* 2021) can be used to automatically code movements. Furthermore, the software can be extended to code facial expressions using an extension known as OpenFace (Baltrusaitis *et al.* 2018), which may overcome the limitations of the present study by providing a more impartial and accurate quantification of behaviours.

### Future directions

Following on from this feasibility study, a randomised controlled trial (RCT) with sufficiently powered sample sizes, is recommended to establish the true effectiveness of the dance-based exergame for enhancing autistic children’s social communication skills using a similar methodology to the one presented here. Overall, the results indicated a small-to-medium effect of the intervention on children’s social communications skills with regard to ERSSQ scores in a small sample size. A power analysis suggests at least 128 participants are needed (with 64 children in each group) to detect a small-medium effect size between an intervention group and a control group with an 80% power and an α = 0.05. Enlisting multiple education providers would support recruitment and guarantee adequate sample sizes are met as individual recruitment for research projects can often be challenging, as found in this feasibility study and reflected by the small home-based sample size. A greater sample size will also afford a deeper exploration into various confounding factors; such as investigating the effect of percentage participation or exercise efforts, familiarity with *Just Dance,* and previous experience of *Just Dance* on social communicative outcomes.

Following on from the thematic analysis of the transcribed semi-structured interviews, future research may ensure children are asked to pre-select their songs before each *Just Dance* session to bolster motivation and ‘buy in’ to the sessions. Likewise, in the school-based studies, teachers employed a ‘*Just Dance* Champion’ to provide children ownership of the intervention and increase motivation. Similar methods should be included in future studies exploring the use of *Just Dance* in the home and school environments for autistic children. Similarly, to further heighten children’s motivation, a financial incentive could be provided, which again may support recruitment. The calendar-style grids reflected that children were more amenable to completing two-to-three songs in the home-based study and two songs were an appropriate session duration in the school-based study. Therefore, it is proposed that the structuring of the intervention in future home-based studies is adapted to reflect the preferences of participants. For example, completing three songs each session rather than the initially proposed five songs. Finally, multiple informants are encouraged to gain a greater and more well-rounded insight into children’s social communication skills. This will also help to reduce the likelihood of a rating bias and provide a greater overview of children’s emotional and social skills. For example, Beaumont *et al.* (2021) employed the use of the ERSSQ-P and ERSSQ-T in a single study to explore the generalisability of their results as observed by parents and teachers of the child. The data was collected at the same time points and analysed in isolation. A similar methodology could be employed in future studies to provide a greater understanding of children’s social competence from different informants.

## Conclusion

Overall, the results from this multiple location feasibility study suggest that *Just Dance* is a fun and enjoyable exergame that can be implemented into different environments with relative ease. Furthermore, *Just Dance* may positively influence autistic children’s social communication skills through the successful inclusion of mirroring, attendance to rhythmical stimuli, and affording an opportunity for interpersonal motor synchrony on the virtual platform. Additional research is needed to ascertain the true effectiveness of participation in *Just Dance* for autistic children’s social-communication skills through adequate sample sizes and comparison groups. Nonetheless, the present feasibility study advocates that *Just Dance* is an engaging physical activity that children and parents and/or children and teachers can participate in together, within their respective environments. Furthermore, the exergaming platform affords children a variety of choices within the intervention; ensuring it is person-centred and allowing individuals to have ownership over the activity.

## Supplementary Material

Supplemental Material
