# Supplementary material for: Exploring the use of a dance-based exergame to enhance autistic children’s social communication skills in the home and school environments: a feasibility study
Source: Int J Dev Disabil. 2023 May 22;71(1):141–58. doi: 10.1080/20473869.2023.2212985 (PMC11774173; doi:10.1080/20473869.2023.2212985)
Supplement: Supplemental Material [file YJDD_A_2212985_SM9630.docx]

**Supplementary Material**

### Supplementary Table 1. Domain summaries, themes, and sub-themes extracted from thematically analysing the transcribed parental exit interviews. Exact quotes and frequencies at which themes and sub-themes were referenced are also provided.

| Domain Summary  Theme  Sub-theme | Quotes | Frequency of References |
| --- | --- | --- |
| 1. Appreciation for the organisation and the information provided throughout the project. | | |
| Parents appreciated the information provided | “There wasn’t really any point where I was like struggling to understand what I was meant to do” – Parent B | 18 |
| Parents appreciated the hybrid approach to training | “Oh, the webinar was awesome yeah and also super helpful, so was the training manual like I think it was very detailed. I mean, there was a lot of information conveyed” – Parent C | 2 |
| Parents liked the training manual | “I mean it looked very, very nice, as well as. The way it was put together, very easy to navigate… It’s definitely something I found useful” – Parent A | 12 |
| Parents enjoyed the training webinar | “I think the webinar was nice because it was interactive and you could ask questions, you can see, you know, like the individuals and stakeholders of the project face to face, so to speak. That was very good” – Parent C | 7 |
| Attendance of recruitment webinar | “That's the first one...? Yeah. Yep. Yeah, I did come to that one... I think it was useful, yeah” – Parent D | 8 |
| Parents valued the organisation of the project | “But I mean speaking specifically to how the project was organized and presented and so on, I think you did a spectacular job like, and I mean I've seen quite a few, you know research projects because I've worked in a University in research for a while, so I’ve seen you know a lot of you know, research projects put together and like just like the care for the visual and visual presentation, like everything from typography to the general design of all the material that you put together, I think, was impeccable” - Parent C  “I say it was pretty straightforward to the instructions and the fact that there was a bit of a build-up so you were gently introducing us to give us as an idea of what would be happening, and that was useful too because you kind of had an idea, about what you were doing at the next stage, like ‘right, this is what we're going to be doing’” – Parent B | 14 |
| Keeping in touch and the researcher being available | “You kept in touch with us and on a regular basis and we knew, well I knew, that I could contact you if there was any problem at any time” – Parent B | 6 |
| 2. Importance of preparing children and offering options before and during the intervention | | |
| Giving children options or control of the intervention | “Having a sense that he can actually choose amongst several options is really important to him, and then knowing what's going to come in, in advance, is also really soothing and comforting to him” – Parent C  “Yeah, for her to be more in control of the tasks that she's doing; she thrives off that, if she’s in control…When we changed it round and got her to do be more in control that definitely had an effect because she was more willing to take part” – Parent B | 16 |
| Praise and feedback are important for children | “You're getting the feedback as well from it it's giving you the stars; as I say, she's very motivated by praise and everything, so that keeps her going” – Parent B | 4 |
| Children appreciate feeling prepared | “Only thing with him is. If you try to do that something for the first time or even second time, it's difficult. After that he enjoys starts enjoying it” – Parent D  “I tried to prepare her and to say, “look we’re going to do thing, we’re going to do some dancing before it even started, so I was trying to get her prepared” – Parent B | 14 |
| 3. Personal circumstances affecting the delivery of intervention | | |
| Family commitments affecting delivery of the intervention | “For me, because I had that big holiday booked in, I was like Okay, I signed up for this but now I’m not sure I can do it because we are away for such a long time, but yeah it did work eventually yeah” – Parent A | 9 |
| Scheduling of intervention was perceived as feasible | “I think so. They were okay. I believe it's two sessions again, you know, it depends on the time” – Parent D  “I think two days a week, it is more now - it should be feasible, it should really yeah I think it's okay, the two a week” – Parent A | 4 |
| Creating structure was important for children | “Right. So, I mean, I'm just talking about myself more generally. It's better to have things with him every day, or like repetitive, you know, then he starts enjoying it… I mean, for them, an autistic child, it's that kind of like the routine, right. If you do something out of routine, they don't generally like it, but then slowly and gradually, when things you do more consistently every day, they may start liking it” – Participant D | 3 |
| Doing the intervention on a weekend | “We started off on the weekend and then yeah it's just our schedule really”– Parent A  “Again, it's really weekend-based that we did the work for the project” – Parent C | 5 |
| 4. Feasibility of outcome measures used within the project | | |
| Outcome measures were easy to use | “I mean, the activities were okay to record, and the questionnaire, I think so in terms of completion, yeah it was fine” – Parent D  “As far as I remember, the questionnaire that was very easy” – Parent A | 13 |
| Parents and children liked the calendar style-grid | “Yeah, but it was easy to fill in and all that was straightforward, it's quite good as well to have the visual side of things; you know, having the red, amber, green faces” – Parent B | 2 |
| Consent forms were easy to complete | “Well yeah that was fine, that was easy, I’ve done it many times with other project and it was very similar to everything else” – Parent A | 3 |
| Importance of individualised responses to questionnaire | “The questionnaire, some of them might not be kind of relevant for my son. So, I just kind of, you know, did not answer or maybe. answered. It wasn't very accurate, but of course, you know, everyone has a different way, right” – Parent D | 2 |
| Losing calendar | “I think I've been, to be honest, I lost it actually pretty early in when we started. So. Yeah. I was not able to follow that one. What I was doing was just, I was writing the songs or something on a piece of papers or a diary or something” – Parent D | 2 |
| 5. Positives to take away from the project | | |
| Parents and children enjoyed the intervention and wish to continue | “She just wanted to carry on and she wasn't really interested in the dancing and practicing the dancing, she was just more interested having a carry on with me. But that's still a positive thing you know, she was still having a play around with me” – Parent B  “Yeah, so I’m very happy to continue yes. Okay, so we got the game, we can do it any day” – Parent A | 24 |
| Parents found the intervention easy to use | “Once you get into the process everything you know becomes pretty easy and it's not too onerous and so” – Parent C | 4 |
| Parents valued the importance of physical activity and end goal of the intervention | “I mean your idea of movement and the physicality is, I think it is, it is very, very important.” – Parent A  “Have a craving for communicating and sometime they don't have the tools or the knowledge to do so and expressing themselves bodily through dancing I think is a great outlet and it's a great idea” – Parent C | 7 |
| Parents wanted to do something with their child | “So, I thought, you know, it probably helps me as well to learn something and may be an enjoyable experience with my son” – Parent D | 3 |
| 6. Challenges to the intervention | | |
| There were negative aspects of the intervention | “They [the dances] are way too fast. Even when I was trying to do it, they were too fast….so that was a kind of challenging thing for me and Child D” – Parent D | 12 |
| Children were already tired or potentially burnt-out | “She's getting to just get overworked or overloaded… She’s like ‘I just want to do this; I don’t want other people telling me what to do’” – Parent B  “Yeah, because he shattered, he is tired mentally”. – Parent A | 7 |
| Associating home time with down time | “The problem that I’ve kind of had with it with her, is that Child B associates home time with her kind of down time, when she can kind of do what she wants” – Parent B | 2 |
| 7. Potential improvements and changes for future studies | | |
| Being there (virtually) for the first session | “If you have a one or two sessions, you know, where you can guide the parent and child, you know, like either even virtually on a zoom meeting or something like that for the first session if they get stuck on what is expected” – Parent D | 3 |
| Creating a social story | “You could create social story to try to explain what is happening and why you know there's a change in a routine and so on” – Parent C | 3 |
| Differing from five songs | “She didn’t want to do the five songs. Three songs were enough for her” – Parent B  “Five song is quite a lot, even for me. I’m very unfit so yeah, I think, so I think it's quite a lot because yeah normally, as I said that we do three or four song that's well over 10 minutes, for example. Yeah, I think five is about five was a bit too much” – Parent A | 6 |
| Doing things differently and suggestions for improvement | “Because like, how do I say this, the selection… some of the work and time was spent selecting the songs before the actual session started. You could have some kind of tool to help the children select the songs ahead of time” – Parent C  “Suggest to parents that you could make the video slower so that they're easier to follow as some steps are quite complex” – Parent D | 10 |
| Providing more information | “The only thing it was it wasn't really emphasized, but maybe you didn't want to emphasize it was that it was quite a short time do quite a lot of sessions” – Parent B | 10 |
| Providing an incentive | “But if you could give the kids like a five-pound voucher… a little bit of reward at the end for them” – Parent A | 5 |
| Small time-scale was challenging | “I honestly, I don't think that such a small-time window will generate measurable differences, I know we got there but it might vary, but what I mean is, like, I think, for an intervention to like you know make noticeable differences…Like you know six weeks is, you know, maybe not sufficient for children” – Parent C | 2 |

### Supplementary Table 2. Domain summaries, themes, and sub-themes extracted from thematically analysing the transcribed staff exit interviews. Exact quotes and frequencies at which themes and sub-themes were referenced are also provided.

| Domain Summary  Themes  Subthemes | | Quotes | Frequency of references |
| --- | --- | --- | --- |
| 1. Appreciation of collaborative planning, organisation, and information provided throughout the project. | | | |
| Staff appreciated the research teams’ quick responses | | “I know that as soon as something was passed your way it was dealt with very quickly and sent back and any concerns or queries were answered very quickly so. You know, I think it worked well in that sense, there was no like waiting for anything, it was just done ASAP” | 2 |
| Staff felt they were provided with sufficient information and prepared to start the project | | “Yeah, I did, I definitely feel like I had enough information provided”  “I think we had everything, you know I mean, it was all explained, and we had a list of songs to choose from”  “And yeah, I did feel prepared to go ahead with the project” | 10 |
| Collaborative planning to ensure success of project in school environment | | “Because obviously you came to us with an idea of you know engagement with parents and we said all let’s try and do it with in school and it worked really well, so I feel like it was nice to feel like our students got accommodated” | 3 |
| Staff felt that project was well organized and streamlined | | “No, I wouldn't say from your point of view of how you've organized at all, there weren’t any bad points”  “Yeah, I mean, it was pretty straightforward” | 6 |
| Project liaisons were able to train other members of staff in delivery of the intervention and completion of outcome measures using information provided | | “Yeah, I just briefly wrote down, I think I bullet pointed basically what you'd sort of relayed to us and, like the reasons behind it and, like sort of what you expected or not expected, but what the research project was and then just gave them the information I think they needed”  “Once I got the time to sit down and almost like kind of train the staff into what we wanted the sessions to be like and how we wanted it to go ahead, then it seemed to flow quite easily then” | 7 |
| 2. Importance of creating structure and routine both within the sessions and throughout the intervention. | | | |
| Doing the intervention daily helped to create structure and routine | | “It was just a case of getting ahead of ourselves and building it into the day, every day, you know what I mean, the two songs”  Yeah, I mean definitely doing it daily is something that students from our school would need and so it's incorporated into a routine | 3 |
| Children’s expectations should be managed to help limit any anxieties | | “Yeah, so we have a visual timetable, so I knew that, well I always use to put it in where I planned to have it [the two songs] for that day. So, yeah, they always knew it was coming in general and when to expect so that was how we tried to create structure” | 2 |
| Using the same songs every day to help manage expectations | | “I also had some comments from some teachers who wanted to be involved and that they feel like the same two songs every day would be better for their class obviously that's on an individual basis” | 1 |
| Staff created structure within the sessions using different songs | | “When the first one [song] was a little bit higher and then the second [song] is a little bit lower, that cued the children in that it was going to finish”  “We queued in, like, we always sing with them just a simple song ‘it's finished it's finished’, so they know that it's done and that kind of cued them into know that it was done” | 8 |
| 3. Emphasizing the individual-ness of each child within the classroom. | | | |
| Staff adapted the protocol to be more individual to each child | | “Because everyone's on such an individualized timetable if it was appropriate to be a little bit later in the day, then that’s just how we played it out and it and it did work well”  “Sometimes it'd be just a case of lowering the lights or pulling the blinds down and they engage with just the music and a bit of the visual and so yeah, we it was easy to adjust to it was it was a successful project in that sense”  “So yes, sometimes it was, we would do it more when it slotted in really so we may do it to breakup English and math sometimes. Other times we would do it at the start of the day or the start of the afternoon and do two songs together. We didn't particularly always do it at the same time every day, we just built in whenever it worked for that day and the children if you see what I mean” | 12 |
| Staff supported individual participation; not forcing participation and allowing each child in to join in how they would like | | “We did sort of go for one child – one chance for everyone to join in if you see what I mean so no one felt pressured. You know I mean, with that sort of activity, if they couldn't access it at the time or they didn’t want to then we didn't come back to it, and if they could you know I mean we worked with it and then moment”  “One of the young men, some days he would sit in and be involved in his own way, but other days he'd just keep going in and out and in and out of the classroom to the music and well that was his way of participating” | 9 |
| Staff highlighted the ‘individual-ness’ of each child | | “Some of them, you know, some of them, the dancing sort of was a way of getting them active but didn't suit some of them. But then again, for most of them it was perfect. I suppose, in any activity, you have some it works for some and it doesn't work for”  “Yeah, we had the enthusiastic, bubbly, confident ones at the front and then just more quiet ones that joined in as and when they wanted to and just did bits and pieces with us at the back of the classroom” | 8 |
| Working to the children's schedules | | “Definitely its beneficial to have that kind of flexibility, I think, to fit it in and say ‘we must do it now’ or ‘we must do it at such and such at time’ that doesn't always feel right, I mean like some, for example, sometimes they’re down and tired and needed calm time and then other times we had to appreciate that they were down and tired and we needed to pick them up, so we would use it then and work with the children” | 5 |
| 4. Benefits of participating in the intervention and positives of the project, including ease of implementation and flexibility. | | | |
| Highlighting the benefits of movement and being active | “Yeah, I thought it was really interesting and I think it made me more aware of how you should keep children active”  “Movement can try to help self-regulate so I definitely think there is potential benefits”  “Some of them it suited some of them it didn't, I mean the activeness, as such, suited all of them or I feel being active is really important to build into the day” | | 8 |
| Continuing with intervention after the project finishes and recommending to other schools or classes | “Yeah, I think we definitely will continue to use it, especially on those less active days. It’s in your head now. The kids know it and they know what to expect and they’re familiar with it, so we can just drop back in to it when it is beneficial for the”  “Yeah, yeah, definitely, I’d recommend it to other schools” | | 4 |
| Children enjoy music and music facilitated group cohesion | “They all they all love music, so it, you know, is something that all that, even if they struggle in other activities, through shared music seems to always bring them together in some way so”  “Music seems to always bring them together in some way so” | | 6 |
| Children enjoyed the intervention | | “Yes, so I think the children enjoyed a lot of it” | 8 |
| Intervention may aid learning | | “Yeah, I think, so I just definitely think it is beneficial for children, and I think, and it can definitely help with their learning and stuff” | 2 |
| Staff participating in the intervention to increase children's participation and supported children to take part | | “Just yeah that I think would be the main thing, just us getting involved rather than sitting back, we also sort of made ourselves look silly in front of the”  “No not particularly, other than sort of support those, I mean, those that found it harder or were less motivated to join in, staff tended to support those children more during the sessions”  “I think just us [staff] getting involved with them, we did it with them each session” | 13 |
| Staff wanted to increase awareness of ASD in research | | “What we are trying to do as a trust is say that, well, our students are really complex and really you know, have got loads of different things going on in their lives, and we need to show that also these questions can't always be used because they're not always applicable for our young people and it's important for them”  “So next time something like this is done, someone will think well actually there's a better way of doing this and that's hopefully what we're hoping will come out of it” | 3 |
| Teachers appreciated that the structuring of sessions was not concrete | | “And I think it worked really well because you were so flexible with us… as long as we did the two songs we could be as flexible as when and, you know, how we did it” | 3 |
| The intervention allowed for flexibility of the sessions and choice; length, time, songs choices, and environment | | “Yeah, I think everything we do has to be so fluid to fit with the children. And so I think, yeah, you have to make sure everything is fluid and I think we do it dependent on what would work that day”  “I think, so I think there's obviously so many different songs and different styles of music on there, I think… I mean I would hope most children would find at least a couple songs that they sort of would take too. I think you can adapt it quite a lot of depending on like the age group and sort of their ability like the age they’re working at, that sort of think”  “In terms of things that went well, I think were choosing their own songs, obviously that worked really well and I think for us, just giving them the freedom to do as they wished”  “Yeah, so it's that's why it's so good, because it is so flexible, so they still get the same songs as their peers but just in a different environment better suited to them” | 21 |
| Two songs were an appropriate duration for each session and was easy to implement | | “Yeah, they did the two songs every day”  “From the teachers I have spoken to the two song I think the two songs was absolutely fine”  “I think the two songs I think that's good because it sort of gives me and them time to sort of get into it” | 13 |
| The outcome measures were easy to complete | | “I think the questionnaire was good, I think that how it was made and the formatting of it was easy to use and yeah, I think that will have made the process easier for some people” | 4 |
| Answering questions on the questionnaire as honestly as possible | | “I just took the questions of the questionnaire at face value and sort of answered them as honestly, as I could, at that moment, you know what you mean” | 3 |
| 5. Challenges to the intervention and drawbacks of the project. | | | |
| Dancing was not some children’s “thing” | | “Some of the students did struggle with staying in the room, with all of their peers doing it at once”  And I think what didn't work is purely for some of them dancing was just not their style of activity, you know, it just perhaps wasn’t suited to them | 6 |
| Songs were too fast or overwhelming for some children | | “I mean for a couple of them, the music was sometimes a bit loud but that was something that we could adjust” | 3 |
| Items on the questionnaire need to be responded to on an individual basis and limit the literal interpretation of the items | | “I have quite, like, an abstract view of stuff… So, when I might read the questions and it was like…One of them was “Does the student start conversations with other students” from the position I'm in I might take that quite differently, as to what a TA might think, or some of the teams may have taken that in quite a literal sense”  “We need to show that also that these questions can't always be used because they're not always applicable for our young people and it's important for them” | 3 |
| Difficulties completing the questionnaire and parental concerns raised regarding the validity of the questionnaire items | | “One parent, who’s child was involved, wanted me to go through the questions and was a bit like ‘oh I don't really understand how you're going to get feedback from this’, so it just took a conversation from me, for them to think right, we need to think of this in a broader sense…”  “The questions were quite hard and I think, and they would have to be tweaked for if you did it further, or you know the study went on further, and in that sense” | 4 |
| Staffing issues (limited staffing) affecting completion of outcome measures | | “Obviously due to staffing issues which is out of everyone's control, we couldn't go ahead with it as soon as we would have liked competing the questionnaires”  “Yeah, but I think that's been the hardest thing for me is getting the teachers to complete them” | 9 |
| Time constraints affecting delivery of intervention | | “I think, so I think possibly not always twice a day or every day, as you know sometimes if we’re out like for half the morning and have to try and fit it in, I mean if we’ve already been doing something active” | 3 |
| 6. Adaptations to the protocol during delivery and suggestions for future studies. | | | |
| Enhancing motivation to take part | | “Well, what seemed to be working well in there, they said they'd pick their first song, and then they do like a ‘just dance champion’ so then whoever sort of was participating the most then got to choose the next song”  “I suppose, them [the teachers] modelling it themselves and joining in probably helped children to actually join in and, I’m trying to think of what else, like letting the children pick the songs so it was all songs they were interested in” | 5 |
| Children took ownership of the sessions | | “So, they really enjoyed that freedom of choosing that one, you know the songs that they liked and I think that was a real key in keeping them focused, you know”  “Yeah, I think, so yeah, definitely having ownership, so it did become very much their sort of sessions that we helped to manage or run it and were like here you go” | 5 |
| Staff were able to overcome any challenges | | “I say if it was that it was too many, with the six students, in the room in the one class, we would split it up into different groups and that worked quite well” | 4 |
| Creating visuals for sessions | | “I would probably make symbol work lists or meet visuals to go along with it” | 3 |
| Selecting staff members who have the capacity to complete the outcome measures | | “So maybe if you have to do with another school, maybe the school could pick teachers, that they would maybe know that it would be more streamline” | 1 |
| Targeting older students | | “It'd be interesting if we could do it, maybe with it is difficult, but with the post-16 students…because there's quite a lot of students, the older students who would, I think, really benefit from it quite a lot” | 1 |
| Targeting other students | | “I think, maybe, maybe like in the future if you ever thought about doing it with different groups of people, obviously, we have like PMLD students and we have an MSI students too who may benefit” | 1 |
